# Tubular epithelial cell-to-macrophage communication forms a negative feedback loop via extracellular vesicle transfer to promote renal inflammation and apoptosis in diabetic nephropathy

**DOI:** 10.7150/thno.63735

**Published:** 2022-01-01

**Authors:** Wen-juan Jiang, Chuan-ting Xu, Chang-lin Du, Jia-hui Dong, Song-bing Xu, Bing-feng Hu, Rui Feng, Dan-dan Zang, Xiao-ming Meng, Cheng Huang, Jun Li, Tao-tao Ma

**Affiliations:** 1 Inflammation and Immune Mediated Diseases Laboratory of Anhui Province, Anhui Institute of Innovative Drugs, School of Pharmacy, Anhui Medical University, Hefei, 230032, China.; 2 The Center for Scientific Research of Anhui Medical University, Hefei, 230032, China.

**Keywords:** diabetic nephropathy, extracellular vesicles, leucine-rich α-2-glycoprotein 1, tumor necrosis factor-related apoptosis-inducing ligand, death receptor 5, tumor necrosis factor α, interleukin

## Abstract

**Background:** Macrophage infiltration around lipotoxic tubular epithelial cells (TECs) is a hallmark of diabetic nephropathy (DN). However, how these two types of cells communicate remains obscure. We previously demonstrated that LRG1 was elevated in the process of kidney injury. Here, we demonstrated that macrophage-derived, LRG1-enriched extracellular vesicles (EVs) exacerbated DN.

**Methods:** We induced an experimental T2DM mouse model with a HFD diet for four months. Renal primary epithelial cells and macrophage-derived EVs were isolated from T2D mice by differential ultracentrifugation. To investigate whether lipotoxic TEC-derived EV (EV_e_) activate macrophages, mouse bone marrow-derived macrophages (BMDMs) were incubated with EV_e_. To investigate whether activated macrophage-derived EVs (EV_m_) induce lipotoxic TEC apoptosis, EV_m_ were cocultured with primary renal tubular epithelial cells. Subsequently, we evaluated the effect of LRG1 in EV_e_ by investigating the apoptosis mechanism.

**Results:** We demonstrated that incubation of primary TECs of DN or HK-2 mTECs with lysophosphatidyl choline (LPC) increased the release of EV_e_. Interestingly, TEC-derived EV_e_ activated an inflammatory phenotype in macrophages and induced the release of macrophage-derived EV_m_. Furthermore, EV_m_ could induce apoptosis in TECs injured by LPC. Importantly, we found that leucine-rich α-2-glycoprotein 1 (LRG1)-enriched EV_e_ activated macrophages via a TGFβR1-dependent process and that tumor necrosis factor-related apoptosis-inducing ligand (TRAIL)-enriched EV_m_ induced apoptosis in injured TECs via a death receptor 5 (DR5)-dependent process.

**Conclusion:** Our findings indicated a novel cell communication mechanism between tubular epithelial cells and macrophages in DN, which could be a potential therapeutic target.

## Introduction

Diabetes mellitus is a group of metabolic disorders characterized by high blood sugar levels over a prolonged period 1. Type 2 diabetes mellitus (T2DM) has the greatest impact and accounts for approximately 90-95% of the global diabetes burden 2. Complications associated with T2DM include heart attack, kidney failure, unhealed wounds, vision loss, and nerve damage 3, 4. Furthermore, the rapid global spread of COVID-19 has caused millions to suffer 5-7. The concurrent global pandemics of COVID-19 and T2DM have resulted in T2DM being the second most common comorbidity of COVID-19 6.

Tissue stress or dysfunction induces inflammation, which helps tissues adapt to noxious conditions and restore tissue functionality. These processes also include a change in the state of cells from stressed to apoptotic 8. Diabetic nephropathy (DN), a serious renal complication of T2DM, is characterized by renal tubular epithelial lipoapoptosis (i.e., apoptosis induced by toxic lipid mediators) and inflammatory cell infiltration that is represented, in part, by activated macrophages 9, 10. However, in DN, it remains unclear how TEC injury promotes macrophage activation and how macrophage activation influences injured TECs and stimulates injured cells to undergo apoptosis to maintain homeostasis. Damaged cells send signals to activate macrophages, which release proinflammatory cytokines and thus stimulate injured cells to undergo apoptosis. This cell communication is an essential component in mammalian development and preservation of homeostasis, ensuring fast and efficient responses to alterations or threats within the environment surrounding host cells. Beyond classical signaling through cell-cell contact and soluble factors, such as cytokines, inflammatory mediators, metabolites, and hormones, intercellular communication also occurs through cellular release of extracellular vesicles (EVs). This mode of communication has the potential to deliver a particularly diverse array of messages to EV-accepting cells at a level beyond that of soluble factor signaling, since EVs may carry a number of bioactive molecules, surface receptors, and genetic information 11, 12.

EVs have been implicated in modulation of the immune system. Moon *et al*. 13 reported that hyperoxia-induced, lung epithelial cell-derived and caspase-3-enriched EVs activated macrophages and mediated the inflammatory lung responses involved in lung injury. Lv *et al.* 14 reported that macrophage internalization of exosomes from BSA-treated tubular epithelial cells (TECs) led to an enhanced inflammatory response and macrophage migration, constituting a critical mechanism of albumin-induced tubulointerstitial inflammation. The function of EV is complex. Appiah *et al*. 15 studied how gut epithelial cell-derived exosomes released in the intestinal luminal space during sepsis affected mucosal inflammation. They found that intestinal epithelial cell (IEC)-derived luminal EVs carried miRNAs that mitigate proinflammatory responses. How these two types of cells, macrophages and TECs, communicate in DN is still unclear.

In the current study, we provided evidence suggesting that lipotoxic TEC-derived EV (EV_e_) induced the expression and release of proinflammatory cytokines, such as IL-1β and TNF-α, in macrophages and induced the release of macrophage-derived EV_m_. Furthermore, EV_m_ could induce apoptosis in lipotoxic TECs. Importantly, we found that LRG1-enriched EV_e_ activated macrophages via a TGFβR1-dependent process and that TRAIL-enriched EV_m_ induced apoptosis in lipotoxic TECs via a DR5-dependent process. Taken together, tubular epithelial cell-to-macrophage-to-tubular epithelial cell circulation via EV transfer promoted renal inflammation and apoptosis in DN.

## Methods

### Experimental reagents

Lysophosphatidylcholine (Sigma, St. Louis, MO) was dissolved as previously described and used for cell culture studies at a concentration of 20 µM^16^. β-actin, DR5, kidney injury molecule-1 (KIM-1) primary antibodies and goat anti-rabbit or anti-mouse immunoglobulin G (IgG) horseradish peroxidase (HRP) secondary antibodies were purchased from Bioss (Beijing, China). Antibodies against TSG101, CD63, ARF6, calnexin, TRAIL, LRG1, and GAPDH were obtained from Abcam (Cambridge, UK). Human recombinant LRG1 and TRAIL were purchased from Abcam (Cambridge, UK). A Cr assay kit was purchased from Njjcbio (Nanjing, China). An ELISA kit for ALB was purchased from Jymbio (Wuhan, China). The Annexin V-FITC/PI Apoptosis Detection Kit was purchased from Bestbio (Shanghai, China). Cytochalasin D was obtained from APExBIO (USA). PKH67 was obtained from Sigma (St. Louis, MO).

### Mouse model of T2DM

C57BL/6 mice supplied by the Experimental Animal Center of Anhui Medical University were used to establish the T2DM model. All animal experiments were performed in accordance with the Regulations of the Experimental Animal Administration issued by the State Committee of Science and Technology of China. Efforts were made to minimize the number of animals used and their suffering. Animals were maintained in accordance with the guidance of the Center for Developmental Biology, Anhui Medical University, for the care and use of laboratory animals, and all experiments used protocols approved by the institutions' subcommittees on animal care. C57BL/6 mice were induced by a HFD diet (Xietong Pharmaceutical Bioengineering Co. LTD, Jiangsu, D12492) for four months. The HFD diet ingredients are listed in Supplemental Table 1. Seven days before killing, intraperitoneal injection of streptozotocin (STZ, Sigma Aldrich, St. Louis, MO, USA; 100 mg/kg) freshly dissolved in sodium citrate (0.1 M, pH 4.5) was performed. Diabetes was confirmed by a fasting blood glucose level > 7.8 mmol/L.

### Cell lines and culture

HK-2 and mTECs, kindly provided by Prof. Huiyao Lan, were cultured in DME/F-12 (HyClone, Logan, UT, USA) supplemented with 10% (vol/vol) heat-inactivated fetal bovine serum (Merck Millipore, Darmstadt, Germany) at 37 °C in a humidified incubator under 5% CO_2_. THP-1 cells were cultured in RPMI-1640 (HyClone, Logan, UT, USA) supplemented with 10% (vol/vol) heat-inactivated fetal bovine serum (Merck Millipore, Darmstadt, Germany) at 37 °C in a humidified incubator under 5% CO_2_. For all experiments, THP-1 monocytes were differentiated into macrophages using phorbol 12-myristate 13-acetate (5 ng/mL).

### Primary cell isolation and culture

Bone marrow-derived macrophages (BMDMs) were isolated from wild-type mice as previously described 17. Briefly, bone marrow was flushed from dissected mouse tibias and femurs with sterile PBS. Cells were resuspended and plated in RPMI-1640 (Gibco, Carlsbad, CA) supplemented with 10% heat-inactivated fetal bovine serum (FBS), 100 U/mL penicillin, 100 μg/mL streptomycin, and M-CSF (10 ng/mL) (Novoprotein, Shanghai, China) conditioned media. After 3 days, the medium was replaced with the same medium without M-CSF. BMDMs were used for experiments after a differentiation period of 7 days. For real-time quantitative PCR analysis, 3 × 10^5^ cells were plated per well into 12-well plates. The next day, BMDMs were treated with EV in RPMI 1640 medium for 8 h before RNA isolation.

### Extracellular vesicle isolation

Renal primary epithelial cells and macrophage-derived EVs isolated from T2D mice were collected over a 24 h time period in EV-depleted complete medium, which was prepared by overnight centrifugation at 100,000 × g at 4 °C. Unless stated otherwise, EVs were isolated from cell culture medium by differential ultracentrifugation using a modified version of a protocol by Thery *et al*. 17. The collected medium was depleted of cells and cell debris by consecutive, low-speed centrifugation (2,000 × g for 15 min and 16,000 × g for 20 min). The supernatants obtained were carefully collected and centrifuged for 90 min at 100,000 × g at 4 °C. Pellets from this centrifugation step were washed in PBS, pooled, and centrifuged again for 90 min at 100,000 × g at 4 °C. The obtained pellets were resuspended in lysis buffer (see below), PBS solution or RPMI-1640 medium, depending on subsequent experiments. EV solutions intended for cell treatment were sterile filtered through a 0.22 µm syringe filter. Resuspended EVs were either used for subsequent analysis or aliquoted and stored at -80 °C. For isolation of EVs derived from primary cells, a commercially available ExoQuick-TC exosome precipitation solution kit from System Biosciences (Palo Alto, CA) was utilized.

### Electron microscopy

Isolated EVs were fixed in 2% paraformaldehyde in 0.1 M phosphate buffer overnight at 4 °C. The samples were then placed on a Formvar-carbon-coated grid and air dried for 20 min. After being rinsed with PBS, grids were transferred to 1% glutaraldehyde for 5 min and washed with distilled water. The grids were first contrasted with uranyl-oxalate solution and then contrasted and embedded in a mixture of 4% uranylacetate and 2% methylcellulose (1:9 ratio). The grids were air dried and visualized with a JEOL 1400 electron microscope (JEOL USA, Peabody, MA) at 80 kV. For immunogold staining, grids were blocked with 10% FBS for 20 min followed by overnight incubation at 4 °C with primary anti-TRAIL and anti-LRG1 antibodies diluted 1:20 in blocking solution overnight. Next, grids were incubated with secondary antibody for 1 h. In negative control samples, primary antibody was omitted. Samples were then labeled with protein A-10-nm gold for 1 h. The grids were contrasted and embedded with a mixture of 4% uranyl acetate and 2% methylcellulose (1:9 ratio) and observed as described above.

### Nanoparticle tracking analysis

The concentration and size distribution of isolated EVs were assessed by nanoparticle tracking analysis (NTA) using a NanoSight NS300 instrument (NanoSight, Amesbury, UK). EV samples were diluted with PBS at a range of concentrations between 4 × 10^8^ and 8 × 10^8^ particles per milliliter in a total volume of 1 milliliter. Each sample was continuously run through a flow-cell top plate set up to 23.3 °C using a syringe pump at a rate of 25 µL/min. At least three videos lasting 30 seconds documenting Brownian motion of nanoparticles were recorded and at least 1000 completed tracks were analyzed by NanoSight software (NTA 2.3.5).

### Cell EV uptake

HK-2-derived purified EVs were labeled with the green fluorescent dye PKH67 (Sigma) and washed with 100,000 × g spinning. Differentiated THP-1 macrophages were then incubated with EVs (10^10^/ml) for 1 h (with or without 10 μM cytochalasin D pre-Hirsova *et al*. treatment), and their cellular internalization was observed using an LSM880 confocal microscope (Carl Zeiss, Jena, Germany). HK-2 cells were incubated with THP-1-derived purified EVs as mentioned above.

### Flow cytometry

The extent of programmed cell death was detected by flow cytometry (CytoFlex, Beckman Coulter, USA) using an AV-FITC/PI apoptosis detection kit (Bestbio, Shanghai, China). To evaluate the apoptosis level of HK-2 and mTECs, the attached and supernatant cells were stained with 5 µl of Annexin V-FITC and 10 µl of PI in the dark, detected by flow cytometry, and analyzed using CytExpert 2.1 software (CytExpert, Beckman Coulter, USA).

### Assay of caspase-3 activity

The activity of caspase-3 was measured by using a caspase-3 activity kit (Bestbio, Shanghai China) according to the manufacturer's instructions. Assays were performed on 96-well microtiter plates. Ten microliters of protein extract, 90 μl of reaction buffer, and 10 μl of caspase substrate were added in turn. Then, the protein extracts were incubated at 37 °C for 2-3 h. Samples were measured with Multiskan MK3 at an absorbance of 405 nm.

### Transient transfection

A small interfering RNA (siRNA) was used to silence TGFβ-R1 in THP-1 cells. Cells transfected with scramble siRNA were used as controls. Cells were grown in 60-mm dishes and transiently transfected with SmartPool siRNA (5 nM, Hanbio) using LipoFiter 3.0 (Hanbio). Experiments were performed 48 h after transfection.

### Acridine orange-ethidium bromide staining

The apoptotic morphology of treated cells was detected and distinguished by acridine orange-ethidium bromide (AO/EB) fluorescent staining. The staining method was performed by introducing a mixture of fluorescent dyes, acridine orange and ethidium bromide at a 1:1 ratio to treated cells. HK-2 cells and mTECs (2 × 103 cells/well) were seeded in chamber slides. The adherent cells were washed with 200 μl of PBS, and 2 μl of the dye mixture containing ethidium bromide (100 mg/ml) and acridine orange (100 mg/ml) in a 1:1 ratio was placed on each well of the chamber slide. Chamber slides were examined immediately under a fluorescence microscope (Carl Zeiss Axio Vert. A1, Jena, Germany).

### Creatinine assay kits and albumin ELISA kits

Twenty-four-hour urine samples were collected one day before the sacrifice of mice. The concentrations of creatinine (Cr) in urine from C57BL/6 T2D mice were determined by Cr assay kits according to the manufacturer's instructions. The concentration of albumin (ALB) in urine from T2D mice was measured via ELISA using a commercialized protocol.

### Histopathology

Renal tissues of mice were fixed in 4% paraformaldehyde for 24 h immediately following killing, processed for histological examination according to a conventional method, and stained with PAS, Oil Red O, H&E and F4/80. The slides were scored in a blinded manner and deidentified.

### TUNEL assay

Renal cell apoptosis was examined by TUNEL assay using the One step TUNEL Apoptosis Assay Kit from Beyotime Biotechnology (Beyotime, Jiangsu, China). Briefly, cells were fixed with 4% paraformaldehyde in PBS and then exposed to the TUNEL reaction mixture containing TM red-labeled dUTP. Finally, samples were counterstained with 4′,6-diamidino-2-phenylindole (DAPI). TUNEL-positive nuclei were identified by fluorescence microscopy.

### Western blot

Whole extracts were separated by 10 or 12% sodium dodecyl sulfate polyacrylamide gel electrophoresis (SDS-PAGE), transferred to a polyvinylidene difluoride membrane, and incubated with primary antibodies against LRG1/TRAIL/TSG101/ARF6/CD63/calnexin/DR5/β-actin/GAPDH. The membranes were then washed in TBS-Tween 20 and incubated with the corresponding secondary antibodies. After extensive washing in TBS-Tween 20, protein bands were visualized with an ECL chemiluminescent kit (ECL-plus; Thermo Fisher Scientific, Pittsburgh, PA, USA).

### Real-time reverse transcriptase-PCR

Total RNA was collected from kidney tissues, THP-1 cells and BMDMs using TRIzol reagent (Invitrogen). First-strand cDNA was synthesized using a ThermoScript RT-PCR synthesis kit (Fermentas, Pittsburgh, PA, USA) according to the manufacturer's instructions. Real-time quantitative PCR analyses for mRNA were performed using ThermoScript RT-qPCR kits in an ABI Prizm step-one plus real-time PCR System (Applied Biosystems, Foster City, CA, USA). The products were used as templates for amplification using SYBR Green PCR amplification reagent (Qiagen, Valencia, CA, USA) and gene-specific primers. Relative expression levels were calculated according to the standard 2^-ΔΔCt^ method. The forward and reverse primers used for PCR are listed in Table S1.

### Statistical analysis

Data are expressed as the means ± SEM and represent at least three independent experiments. Differences between two groups were compared using the two-tailed Student's t-test. Differences between multiple groups were compared using one-way analysis of variance followed by Student's t-test. Differences were considered significant at P < 0.05. All analyses were performed using GraphPad Prism 5.0 software (San Diego, CA).

## Results

### Lipotoxicity induces the release of tubular epithelial cell (TEC)-derived EV_e_ *in vivo* and *in vitro*

**Figure 1:**
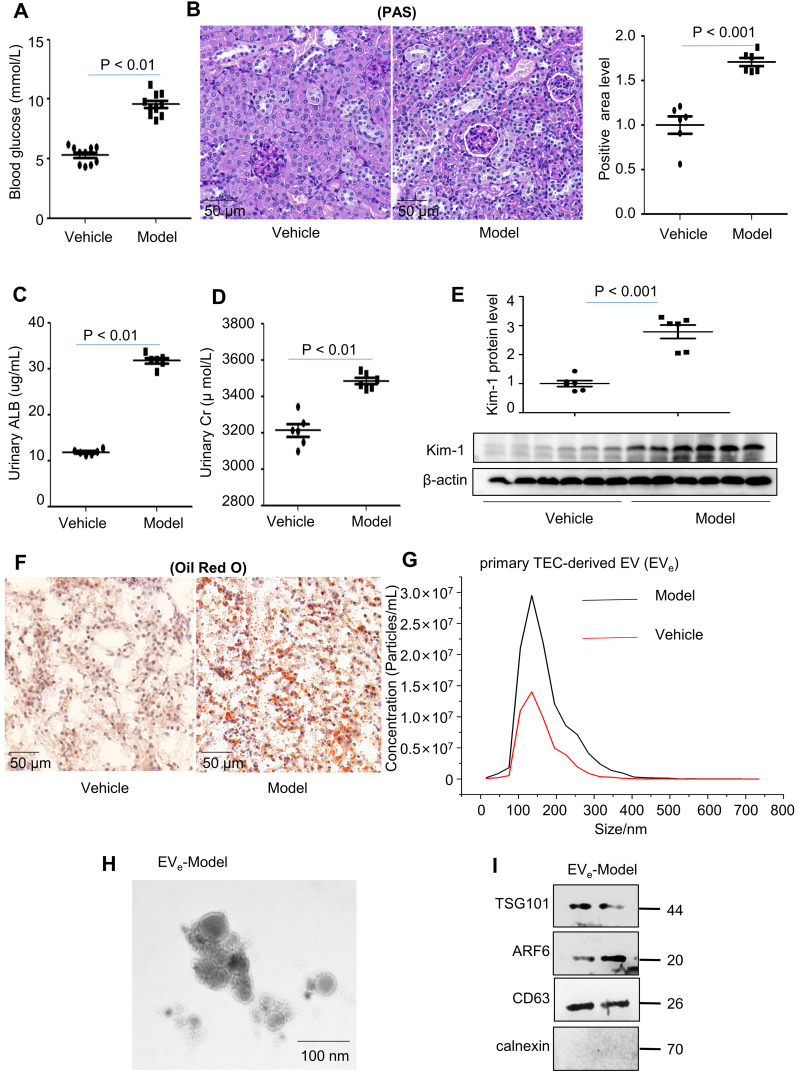
** Lipotoxicity induces the release of tubular epithelial cell (TEC)-derived EV_e_ *in vivo* and *in vitro.* (A)** Blood glucose assay. **(B)** Kidney tissues stained with periodic acid-Schiff (PAS). **(C)** Urinary albumin (ALB) assay. **(D)** Urinary creatinine (Cr) assay. **(E)** Expression of KIM-1 was detected by western blot. **(F)** Kidney tissues stained with Oil Red O (ORO). **(G)** Primary TEC-derived EV_e_ of DN representative image with nanoparticle tracking analysis (NTA). **(H)** Transmission electron photomicrographs of primary TEC-derived EV_e_ of DN. **(I)** The expression of TSG101, ARF6, CD63 and calnexin in primary TEC-derived EV_e_ from DN patients was detected by western blot. Similar results were obtained in 3 independent experiments with 10 mice per group.

To study lipotoxicity in diabetic nephropathy (DN), a mouse model of diabetes was successfully induced by a high-fat diet (HFD) in C57BL/6 mice, as demonstrated by an equal level of increased blood glucose (Figure 1A). Periodic acid-Schiff (PAS) staining showed that glomerular mesangial expansion (GME) was increased in the DN mouse group compared to the vehicle group (Figure 1B). Furthermore, DN mice developed significant urinary albumin (ALB) and urinary creatinine (Cr) at 18 weeks after HFD induction (Figure 1C - D). Immunoblot analysis indicated that KIM-1, a kidney injury marker, was significantly upregulated in the DN group (Figure 1E). Lipid accumulation occurred in tubule cells compared to vehicle, as assessed by Oil Red O (ORO) staining (Figure 1F).

Primary tubular epithelial cell-derived EV_e_ from DN mice were increased by approximately 2-fold, as quantified via nanoparticle tracking analysis (NTA) (Figure 1G). The size characteristics of the released EV_e_ were confirmed by electron microscopy (Figure 1H). Immunoblot analysis indicated that isolated EV_e_ were positive for tumor susceptibility gene 101 (TSG101), adenosine diphosphateribosyltion factor 6 (ARF6) and CD63 and were negative for the endoplasmic reticulum marker calnexin (Figure 1I).

To explore the effect of lipotoxicity on TEC-derived EV_e_ release *in vitro*, we established treatment conditions that did not induce cell death in HK-2 cells and mTECs to avoid the collection of apoptotic bodies. A 6-hour treatment with LPC did not induce apoptosis but increased injury in these cells, and thus they were used for our experiments (Supplementary Figure S1A - B). We then examined the damage from lipids to renal tubular epithelial cells with lysophosphatidyl choline (LPC) to stimulate kidney tubular epithelial cells (HK-2) and mouse kidney tubular epithelial cells (mTECs). As shown in Supplementary Figure S1C - D, the protein level of KIM-1, an established biomarker for renal proximal tubule injury, was increased dramatically after 20 μmol/L LPC stimulation.

Upon LPC treatment, released EV_e_ were isolated from the cell culture medium and quantified via nanoparticle tracking analysis (NTA). Over a 6-hour incubation period, LPC induced an approximately 2-fold increase in the release of EV_e_ in HK-2 cells (Supplementary Figure S2A). The size characteristics of the released EV_e_ were confirmed by electron microscopy (Supplementary Figure S2B). Immunoblot analysis indicated that isolated EV_e_ were positive for TSG101, ARF6 and CD63 (Supplementary Figure S2C). Similar results were confirmed in mTECs (Supplementary Figure S2D - F).

This suggested that lipotoxicity induced the release of tubular epithelial cell (TEC)-derived EV_e_* in vivo* and *in vitro*.

### Lipotoxic TEC-derived EV_e_ activate macrophages

**Figure 2:**
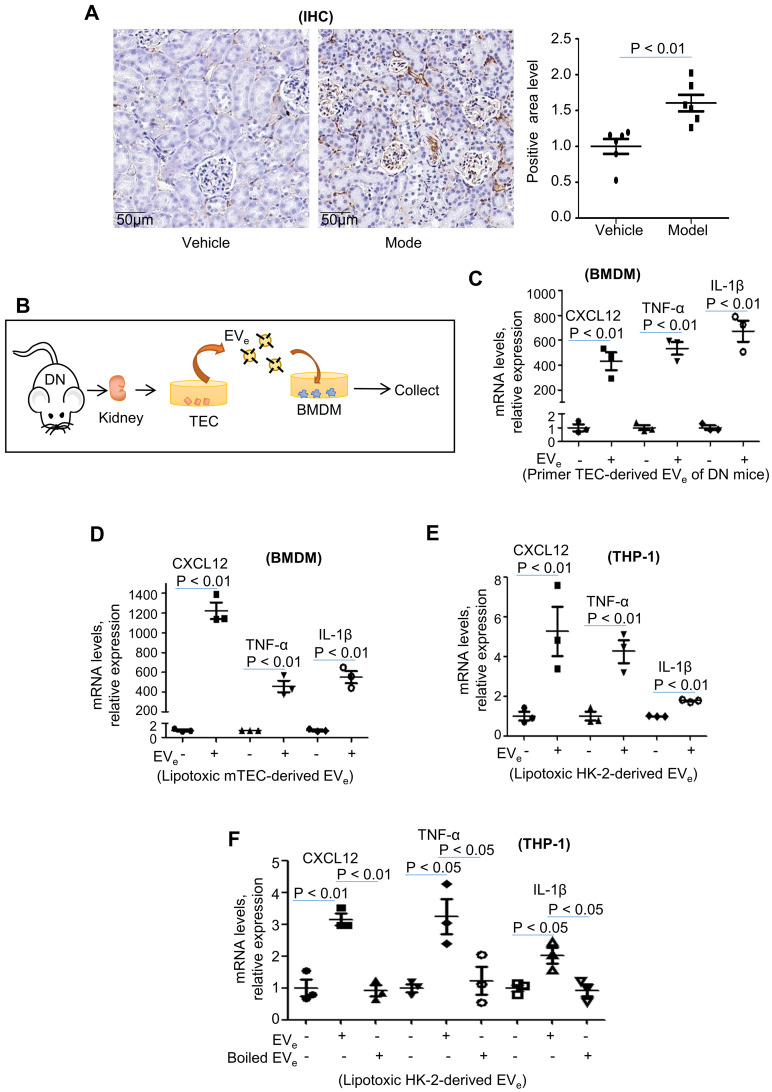
** Lipotoxic TEC-derived EV_e_ activates macrophages. (A)** Immunohistochemical staining of F4/80 in kidney tissues. Scale bar, 50 μM; magnification, 20 ×. **(B)** Mouse bone marrow-derived macrophages (BMDMs) were incubated with purified EV_e_ (10^10^/mL) isolated from DN mouse-derived TECs. **(C)** The levels of CXCL12, TNF-α, and IL-1β in BMDMs treated with primed TEC-derived EV_e_ from DN mice were analyzed by quantitative real-time PCR. **(D)** The levels of CXCL12, TNF-α, and IL-1β in BMDMs treated with lipotoxic mTEC-derived EV_e_ were analyzed by quantitative real-time PCR. **(E)** The levels of CXCL12, TNF-α, and IL-1β in THP-1 cells treated with lipotoxic HK-2-derived EV_e_ were analyzed by quantitative real-time PCR. **(F)** The levels of CXCL12, TNF-α, and IL-1β in THP-1 cells treated with lipotoxic HK-2-derived EV_e_ and boiled lipotoxic HK-2-derived EV_e_ analyzed by quantitative real-time PCR. Similar results were obtained in 3 independent experiments or in triplicate culture assays.

Inflammation is a central contributor to kidney pathology during DN. Characterization of DN mice was confirmed by infiltration of macrophages detected by immunohistochemical analysis. As shown in Figure 2A, the infiltration of macrophages was clearly upregulated in DN mice.

Interestingly, mouse bone marrow-derived macrophages (BMDMs) were incubated with primary tubular epithelial cell-derived EVs (EV_e_, 10^10^/ml) from DN mice (Figure 2B), and EV_e_ significantly increased CXCL12, TNF-α and IL-1β mRNA expression (Figure 2C). Similarly, incubation of BMDMs with LPC-treated mTEC-derived EV_e_ (10^10^/ml) (Figure 2D) or incubation of THP-1 cells with LPC-treated HK-2-derived EV_e_ (10^10^/ml) (Figure 2E) also increased TNF-α, IL-1β, and CXCL12 mRNA expression.

To demonstrate that an EV-unrelated fraction was not responsible for macrophage activation, we destroyed EVs by boiling and observed that this fraction abolished their stimulatory activity (Figure 2F).

This suggested that lipotoxicity-induced EV_e_ promote proinflammatory responses.

### LRG1-enriched EV_e_-mediated macrophage activation is TGFβR1-dependent

**Figure 3:**
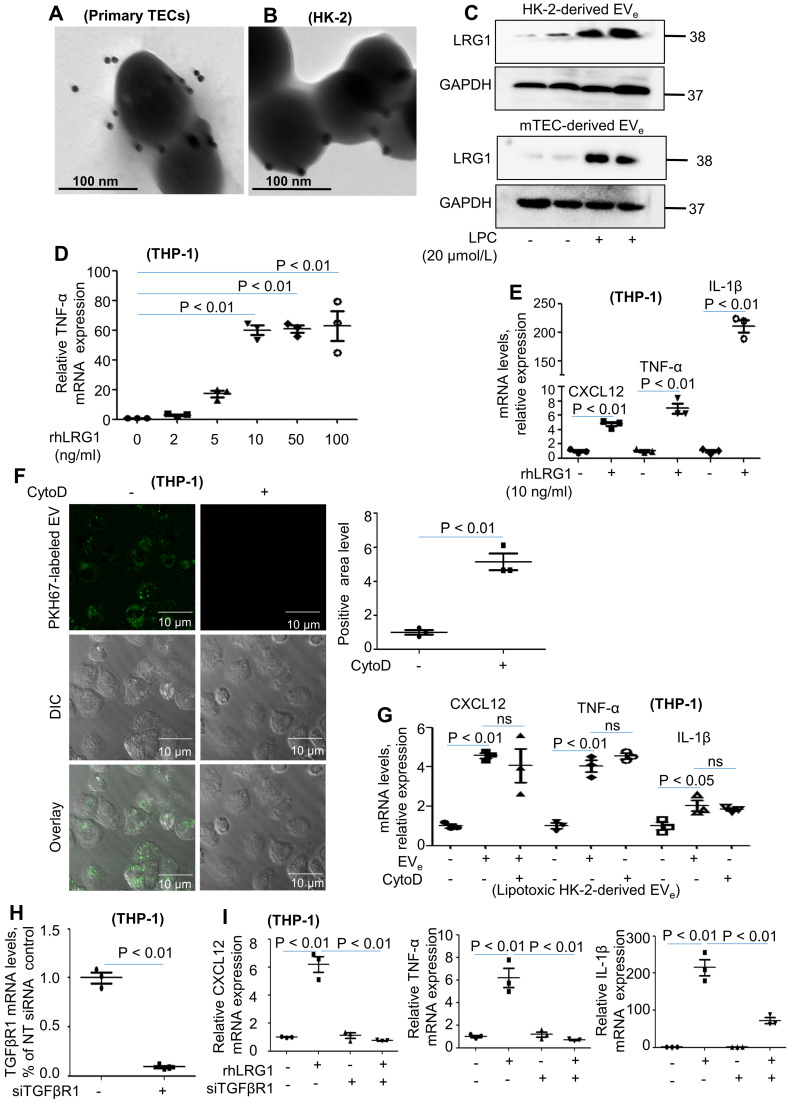
** LRG1-enriched EV_e_-mediated macrophage activation is TGFβR1-dependent. (A)** Representative transmission electron photomicrographs immunogold-labeled with an anti-LRG1 antibody of primary TEC-derived EV_e_ of DN. Scale bar: 100 nm. **(B)** Representative transmission electron photomicrographs immunogold-labeled with an anti-LRG1 antibody of lipotoxic HK-2-derived EV_e_. Scale bar: 100 nm. **(C)** Expression of LRG1 in HK-2-derived EV_e_ and mTEC-derived EV_e_ treated with LPC was detected by western blot. **(D)** Effects of various concentrations of rhLRG1 on TNF-α mRNA levels in THP-1 cells analyzed by quantitative real-time PCR. **(E)** Effects of rhLRG1 (10 ng/mL) on CXCL12, TNF-α and IL-1β mRNA levels in THP-1 cells analyzed by quantitative real-time PCR. **(F)** THP-1 cells were differentiated using PMA treatment for 48 h. Thereafter, THP-1 cells were pretreated with cytochalasin D (1 μM) followed by 2 hours of incubation with EV_e_ (10^10^/mL) isolated from LPC-treated HK-2 cells. Prior to this incubation, EV_e_ were labeled using the fluorescent dye PKH67. EV_e_ uptake by macrophages was visualized using confocal microscopy and quantified. **(G)** Differentiated macrophage-like THP-1 cells were pretreated with cytochalasin D (1 μM) for 1 hour followed by 2 hours of incubation with lipotoxic HK-2-derived EV_e_ (10^10^/mL) for 2 hours. Effect of cytochalasin D on CXCL12, TNF-α and IL-1β mRNA levels in THP-1 cells analyzed by quantitative real-time PCR. **(H)** The efficiency of TGFβR1 knockdown in THP-1 cells analyzed by quantitative real-time PCR. **(I)** CXCL12, TNF-α and IL-1β mRNA levels in THP-1 cells treated with siRNA-TGFβR1 analyzed by quantitative real-time PCR. Similar results were obtained in 3 independent experiments or in triplicate culture assays.

Our previous study revealed that LRG1 was highly elevated upon renal damage 18. Interestingly, immunogold labeling and electron microscopy showed that LRG1 was found in mouse primary TEC-derived EV_e_ (Figure 3A). Similar results were also found in lipotoxic HK-2-derived EV_e_ (Figure 3B). Moreover, western blot analysis showed a significant increase in LRG1 expression in lipotoxic HK-2-derived EV_e_ and lipotoxic mTEC-derived EV_e_ (Figure 3C). In addition, THP-1 cells treated with recombinant human LRG1 (rhLRG1) clearly upregulated the expression of CXCL12, TNF-α and IL-1β (Figure 3D - E). The results indicated that LRG1 induced the expression of cytokines.

We then examined whether phagocytosis was required for macrophage activation by EV_e_. Cytochalasin D, a pharmacologic inhibitor of phagocytosis, efficiently inhibited phagocytosis of fluorescently labeled EV_e_ but had no effect on macrophage activation (Figure 3F - G). These data suggested that EV_e_-macrophage interactions at the level of the plasma membrane were likely responsible for proinflammatory macrophage activation.

Although LRG1 binds directly to TGFβ receptor 1 (TGFβR1) 19, it is not known whether LRG1-enriched EV_e_ could promote the activation of macrophages via TGFβR1. To determine whether TGFβR1 regulated LRG1-enriched, EV_e_-induced macrophage activation, we then treated THP-1 cells with siRNA-TGFβR1 for 24 hours (Figure 3H). PCR analysis indicated that knockdown of TGFβR1 significantly inhibited the expression of TNF-α, IL-1β, and CXCL12 (Figure 3I).

Taken together, lipotoxic TEC-derived, LRG1-enriched EV_e_ promoted the activation of macrophages in a TGFβR1-dependent manner.

### Lipotoxic TEC-derived, LRG1-enriched EV_e_ induce the release of macrophage-derived EV_m_

**Figure 4:**
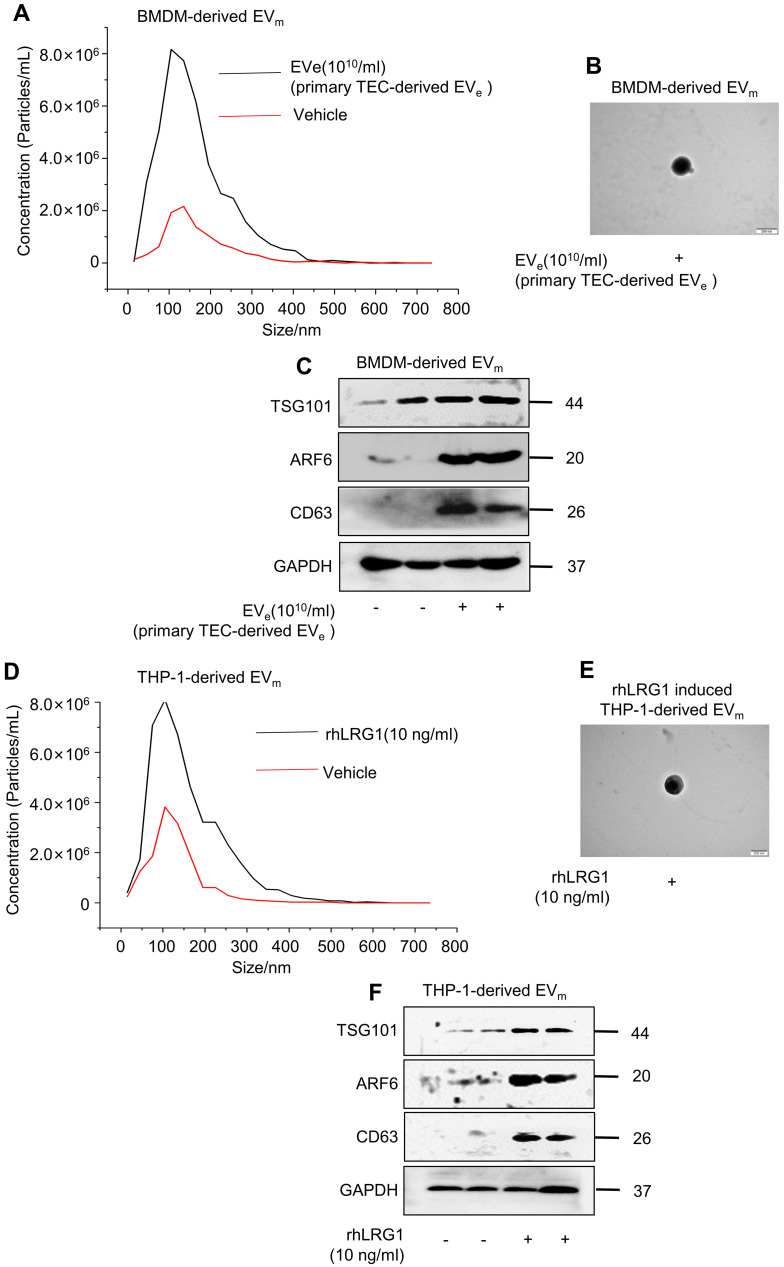
** Lipotoxic TEC-derived, LRG1-enriched EV_e_ induce the release of macrophage-derived EV_m._ (A)** Representative images of BMDM-derived EV_m_ treated with primary TEC-derived EV_e_ from DN with nanoparticle tracking analysis (NTA). **(B)** Transmission electron photomicrographs of BMDM-derived EV_m_ treated with primary TEC-derived EV_e_ of DN. **(C)** Expression of TSG101, ARF6 and CD63 in BMDM-derived EV_m_ treated with primary TEC-derived EV_e_ from DN patients was detected by western blot. **(D)** Representative images of THP-1-derived EV_m_ treated with rhLRG1 with nanoparticle tracking analysis (NTA). **(E)** Transmission electron photomicrographs of THP-1-derived EV_m_ treated with rhLRG1. **(F)** The expression of TSG101, ARF6 and CD63 in THP-1-derived EV_m_ treated with rhLRG1 was detected by western blot. Similar results were obtained in 3 independent experiments or in triplicate culture assays.

Interestingly, we found that lipotoxic TEC-derived, LRG1-enriched EV_e_ not only activated macrophages but also induced the release of macrophage-derived EV_m_. Primary TEC-derived EV_e_ from DN mice induced an approximately 4-fold increase in the release of macrophage-derived EV_m_ in BMDMs, as quantified by NTA in Figure 4A. The size characteristics of the released EV_m_ were confirmed by electron microscopy (Figure 4B). Immunoblot analysis indicated that isolated EV_m_ were positive for TSG101, ARF6 and CD63 (Figure 4C). Furthermore, analysis using NTA, western blot and electron microscopy showed that recombinant human LRG1 (rhLRG1) also induced an approximately 2-fold increase in the release of macrophage-derived EV_m_ in THP-1 cells (Figure 4D - F).

All of the above data supported the conclusion that lipotoxic TEC-derived, LRG1-enriched EV_e_ induced the activation of macrophages and promoted the release of macrophage-derived EV_m_.

### Activated macrophage-derived EV_m_ induce lipotoxic TEC apoptosis

**Figure 5:**
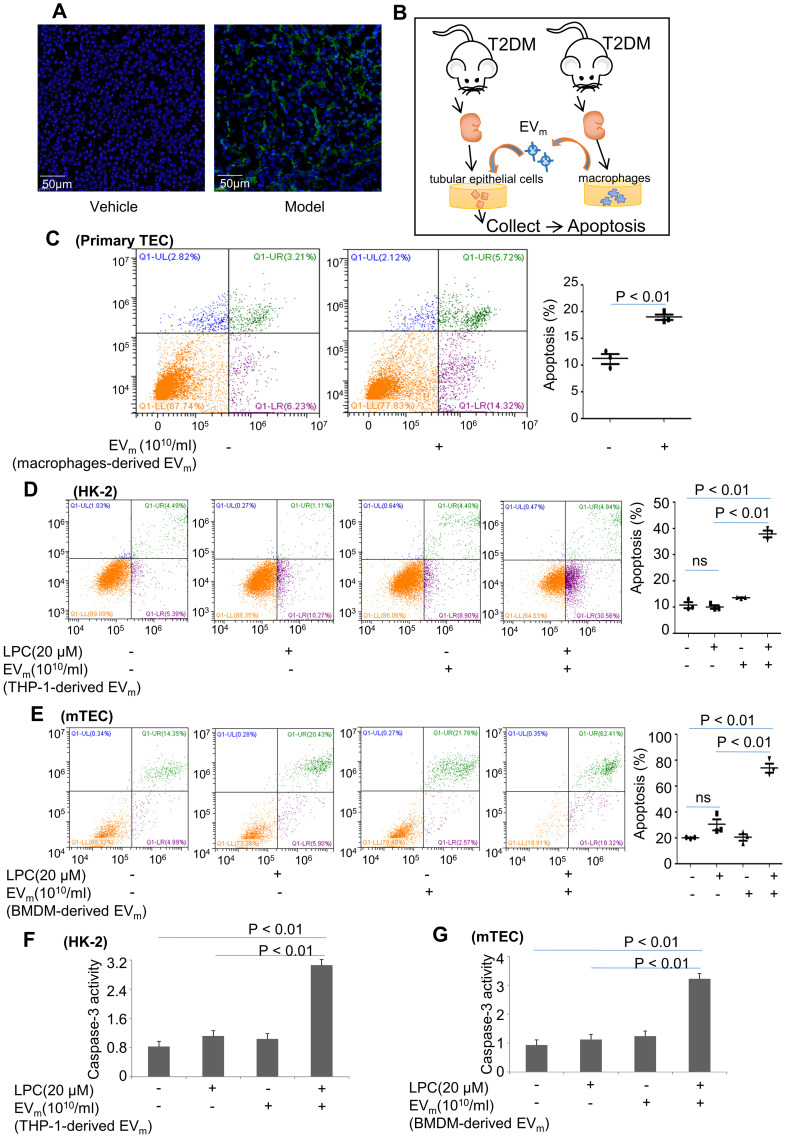
** Activated macrophage-derived EV_m_ induce lipotoxic TEC apoptosis. (A)** Representative images of TUNEL staining in various groups. Scale bar, 50 μm. **(B)** Tubular epithelial cells of DN were incubated with purified EV_m_ (10^10^/mL) isolated from DN mice-derived macrophages. **(C)** Flow cytometry analysis of primary TECs in each group. **(D)** Flow cytometry analysis of HK-2 in each group. **(E)** Flow cytometry analysis of mTEC in each group. **(F)** The activity of caspase-3 in HK-2 cells induced by THP-1-derived EV_m_. **(G)** The activity of caspase-3 in mTECs induced by BMDM-derived EV_m_. Similar results were obtained in 3 independent experiments or in triplicate culture assays.

Available evidence suggests that apoptosis of tubular epithelial cells is one of the characteristics of DN. Numerous TUNEL-positive cells were observed in DN mice in contrast with the vehicle group (Figure 5A).

To confirm the role of EV_m_ in apoptosis of tubular epithelial cells in DN, primary renal macrophage-derived EV_m_ were cocultured with primary renal tubular epithelial cells (Figure 5B). Over an 8-hour incubation period, primary mouse renal macrophage-derived EV_m_ significantly promoted apoptosis in primary renal tubular epithelial cells (Figure 5C).

Next, we treated HK-2 cells with LPC and then cocultured them with macrophage-derived EV_m_ in THP-1 cells. As illustrated in Figure 5D, in the presence of EV_m_, LPC-pretreated HK-2 cells exhibited a much higher level of apoptosis, whereas LPC treatment alone in the absence of EV_m_ did not affect the basal level of apoptosis in HK-2 cells. Similar results revealed that activated macrophage-derived EV_m_ in BMDMs induced lipotoxic mTEC apoptosis (Figure 5E). Interestingly, the activity of caspase-3 in lipotoxic mTECs or HK-2 cells was increased by macrophage-derived EV_m_ (Figure 5F- G).

### Activated macrophage-derived EV_m_ induces tubular epithelial cell apoptosis in a TRAIL-DR5-dependent manner

**Figure 6:**
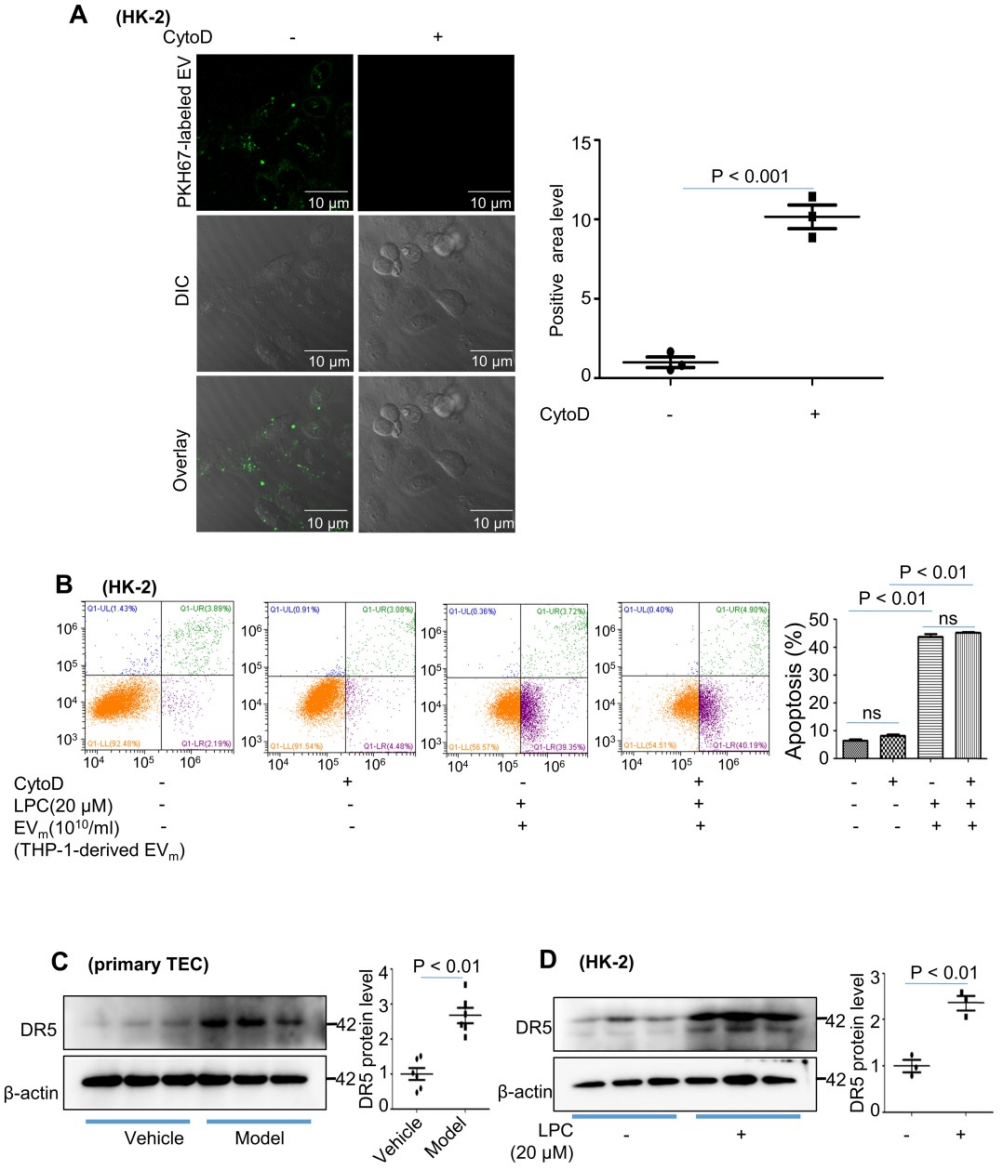
** Activated macrophage-derived EV_m_ induce tubular epithelial cell apoptosis in a TRAIL-DR5-dependent manner. (A)** HK-2 cells were pretreated with cytochalasin D (1 μM) followed by 2 hours of incubation with THP-1-derived EV_m_ (10^10^/mL). Prior to this incubation, EV_m_ were labeled using the fluorescent dye PKH67. EV_m_ uptake by HK-2 cells was visualized using confocal microscopy and quantified. **(B)** Flow cytometry analysis of HK-2 in each group. **(C)** The expression of DR5 in primary TECs was detected by western blot. **(D)** The expression of DR5 in HK-2 cells treated with LPC was detected by western blot. Similar results were obtained in 3 independent experiments or in triplicate culture assays.

We then examined whether phagocytosis is required for tubular epithelial cell apoptosis by EV_m_. Cytochalasin D had no effect on HK-2 apoptosis (Figure 6A - B). These data suggested that EV_m_-tubular epithelial cell interaction at the level of the plasma membrane was likely responsible for tubular epithelial cell apoptosis.

Lorz and colleagues reported that tumor necrosis factor-related apoptosis-inducing ligand (TRAIL)-induced cell death played an important role in the progression of diabetic nephropathy 20. Recent evidence has shown that TRAIL-R2 (DR5) is the protein that is most strongly associated with kidney function decline. We examined the expression of DR5 in primary tubular epithelial cells and found that DR5 was significantly upregulated in the DN group (Figure 6C). Similar results were revealed in HK-2 cells after LPC treatment (Figure 6D).

**Figure 7:**
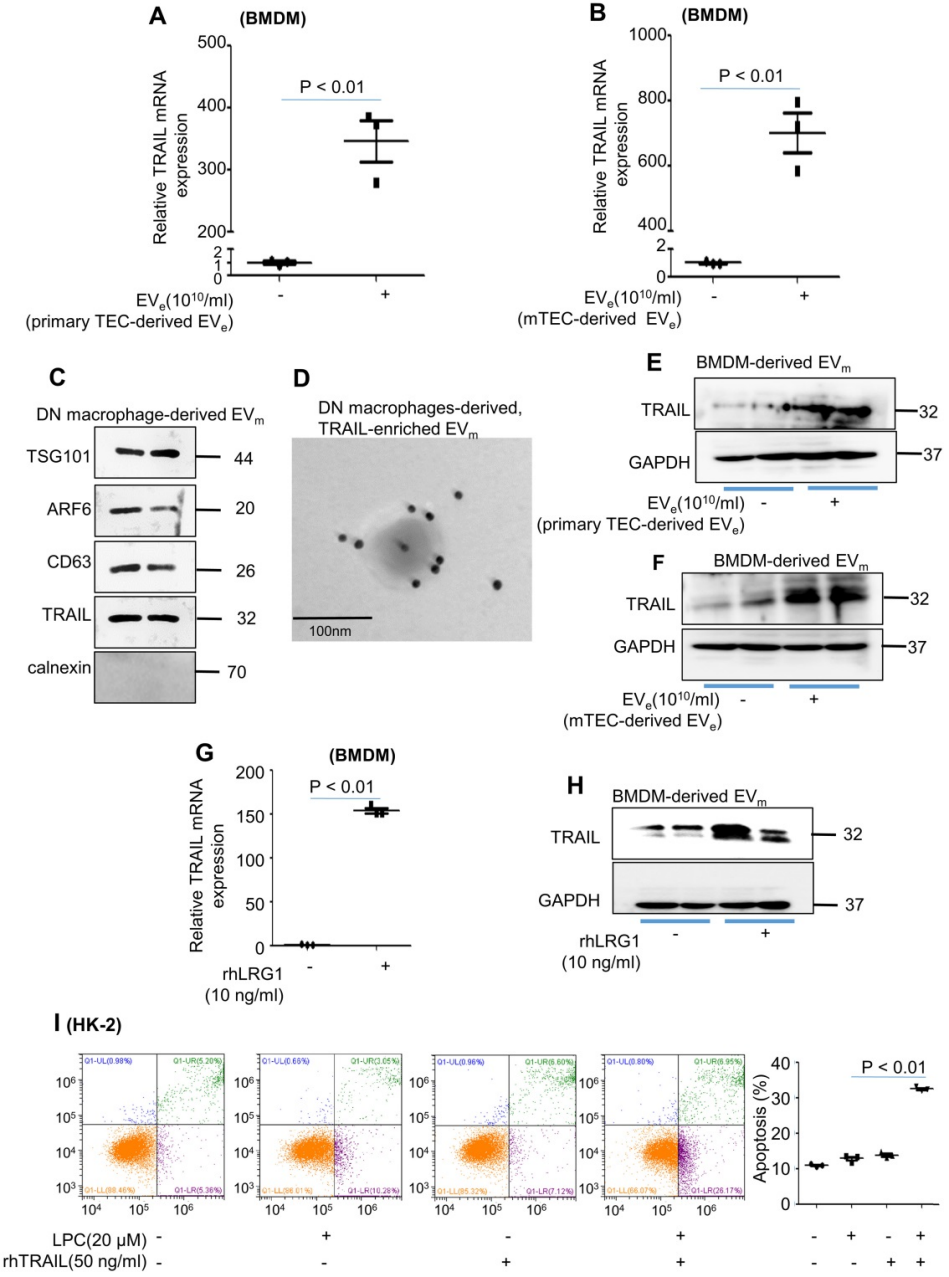
** Activated macrophage-derived EV_m_ induces tubular epithelial cell apoptosis in a TRAIL-DR5-dependent manner. (A)** Effect of primary TEC-derived EV_e_ on TRAIL mRNA levels in BMDMs analyzed by quantitative real-time PCR. **(B)** Effect of mTEC-derived EVs on TRAIL mRNA levels in BMDMs analyzed by quantitative real-time PCR. **(C)** The levels of CXCL12, TNF-α, IL-1β, TRAIL and calnexin in macrophage-derived EV_m_ of DN mice analyzed by western blot. **(D)** Representative transmission electron photomicrographs immunogold-labeled with an anti-TRAIL antibody of macrophage-derived, TRAIL-enriched EV_m_ of DN mice. Scale bar: 100 nm. **(E)** The expression of TRAIL in BMDM-derived EV_m_ treated with primary TEC-derived EV_e_ from DN mice was detected by western blot. **(F)** The expression of TRAIL in BMDM-derived EV_m_ treated with mTEC-derived EV_e_ from DN mice was detected by western blot. **(G)** Effect of rhLRG1 (10 ng/mL) on TRAIL mRNA levels in BMDMs analyzed by quantitative real-time PCR. **(H)** Effect of rhLRG1 (10 ng/mL) on TRAIL protein levels in BMDMs analyzed by western blot. **(I)** Flow cytometry analysis of HK-2 in each group. Similar results were obtained in 3 independent experiments or in triplicate culture assays.

Macrophage infiltration and TEC apoptosis are hallmarks of DN. However, how these two types of cells communicate remains obscure. As illustrated in Figure 7A, primary tubular epithelial cell-derived EV_e_ induced a much higher level of TRAIL, the ligand of DR5, in BMDMs. Similar results were observed in mTEC-derived EV_e_-induced BMDMs (Figure 7B). Interestingly, the expression of TRAIL in macrophage-derived EV_m_ of DN mice was observed by western blot and immunogold labeling (Figure 7C - D). Furthermore, primary tubular epithelial cell-derived EV_e-_ or LPC-treated mTEC-derived EV_e_ induced a much higher level of TRAIL in BMDM-derived EV_m_ (Figure 7E - F). Importantly, rhLRG1 also increased the mRNA level of TRAIL in BMDMs (Figure 7g) and increased the protein level of TRAIL in BMDM-derived EV_m_ (Figure 7H). To gain greater insight regarding TRAIL-induced apoptosis of lipotoxic TECs, recombinant human TRAIL (rhTRAIL) was used. Concomitant stimulation of HK-2 cells with TRAIL and LPC significantly increased apoptosis (Figure 7I).

Taken together, our results indicated that TRAIL-enriched EV_m_ from activated macrophages induced lipotoxic TEC apoptosis via a DR5 proapoptotic signaling pathway.

## Discussion

Diabetic nephropathy (DN), whose pathogenesis is complex, is increasing in incidence and is now the number one cause of end-stage renal disease in the industrialized world 21. Recent studies have confirmed that apoptosis contributes to diabetic nephropathy (DN) 22, 23. Tojo *et al.* 24 reported that tubular dysfunction may partly explain microalbuminuria in early-stage diabetic nephropathy. Kumar *et al.* 25 also found apoptosis in all five DN biopsy specimens examined, either in epithelial cells of the proximal or distal tubules or in endothelial cells or interstitial cells. Mou *et al*. 26 further demonstrated that apoptosis of renal tubular epithelial cells may participate in the development and progression of DN in rats. The potential therapeutic mechanisms of PGE1 and AGEI, at least in part, inhibited the apoptosis of renal tubular epithelial cells. In our study, similar results demonstrated that the apoptosis of proximal tubular cells was increased in a DN mouse model. Cell death via apoptosis is an active response of cells to alter microenvironments 27; however, the mechanism of renal tubular epithelial cell apoptosis is obscure.

Renal lipotoxicity due to derangement in lipid metabolism may be a pathogenic mechanism leading to diabetic nephropathy and renal dysfunction 28. Ectopic fat deposition (EFD) in the kidney has been shown to play a causal role in diabetic nephropathy 29. Opazo-Rios *et al.* 30 found that serum lipid abnormalities and renal ectopic lipid accumulation were associated with the development of kidney diseases, particularly diabetic nephropathy. Xu *et al.* 31 also reported that the manipulation of lipid metabolism might act as a promising therapeutic intervention for diabetic nephropathy. Our study found that LPC promoted proximal tubular cell injury. However, the process by which lipotoxicity promotes the progression of diabetic nephropathy remains obscure.

It has been confirmed that diabetes is an inflammatory disease caused by metabolic disorders. Bohle *et al*. 32 found that infiltration of inflammatory cells was detected by immunostaining in renal biopsy specimens from 488 patients with DN. Inflammation is a complex biological response that is essential for eliminating microbial pathogens and repairing tissues after injury. Macrophages have a crucial role in the regulation of both innate and adaptive immune responses in DN 33, 34. Chow *et al*. 35 reported that kidney macrophages were found to increase with the duration of diabetes, and their numbers correlated with the severity of renal injury and dysfunction. We also found massive macrophage infiltration in the current study. This new pathogenic perspective of DN as an inflammatory condition leads to novel horizons 36. Targeting inflammatory pathways could possibly be a component of the strategies to prevent and control diabetes and related complications 37. However, these processes and mechanisms of activation of macrophages, playing active defense roles and signals transmitted between cells in DN, remain obscure.

Deng *et al*. 38 found that exosome-like vesicles (ELVs) released by adipose tissue can act as a mode of communication between adipose tissues and macrophages. Li *et al*. 39 reported that the HIF-1alpha-dependent release of miRNA-23a-enriched exosomes from hypoxic tubular epithelial cells activated macrophages to promote tubulointerstitial inflammation. Interestingly, we also found that lipotoxicity-induced EV_e_ promote proinflammatory responses. This suggested that lipotoxic TEC-derived EV_e_ activated macrophages in DN. In our previous study, leucine-rich alpha-2-glycoprotein 1 (LRG1) was highly elevated upon renal damage 18. Interestingly, LRG1 was also found in lipotoxic TEC-derived EV_e_. LRG1 can promote completely different cellular processes, including survival, proliferation, migration, and metastasis 40, 41. LRG1 is an innovative biomarker for inflammation and angiogenesis. Liu *et al.* 42 found that LRG1 deletion caused impaired immune cell infiltration. Hong *et al.* 19 identified LRG1 as a potential novel pathogenic mediator that promoted diabetic kidney disease progression by enhancing TGF-beta-induced angiogenesis. In our study, it was also observed that stimulation of TGF-βR1 with recombinant LRG1- or LRG1-enriched EV_e_ activated macrophages toward a proinflammatory phenotype. All of the above data supported the conclusion that lipotoxic TEC-derived, LRG1-enriched EV_e_ induced the activation of macrophages.

Interestingly, we found that lipotoxic TEC-derived, LRG1-enriched EV_e_ not only activated macrophages but also induced the release of macrophage-derived EV_m_. Next, we found that activated macrophage-derived EV_m_ induced lipotoxic TEC apoptosis in DN. To explore the mechanism, we tested the level of DR5 and found that DR5 was significantly upregulated in the DN group and in LPC-treated HK-2 cells. Similarly, Hirsova *et al*. 43 also reported that lipids stimulated the expression of DR5. TRAIL has been studied extensively as a death ligand and initiator of the apoptotic signaling cascade via its cognate receptor DR5 44. Ke *et al*. 45 reported that TRAIL was naturally delivered as a membrane-bound form by EVs and was highly efficient for apoptosis induction. Thereafter, we focused on the potential role of macrophage-derived EV_m_ carrying TRAIL as contributors to TEC apoptosis. It was demonstrated that lipotoxic TEC-derived, LRG1-enriched EV_e_ promoted the level of TRAIL expression not only in macrophages but also in macrophage-derived EV_m_. Herein, we found that stimulation of TRAIL receptor (DR5) with recombinant TRAIL or macrophage-derived TRAIL-enriched EV_m_ promoted TEC apoptosis.

**Figure 8:**
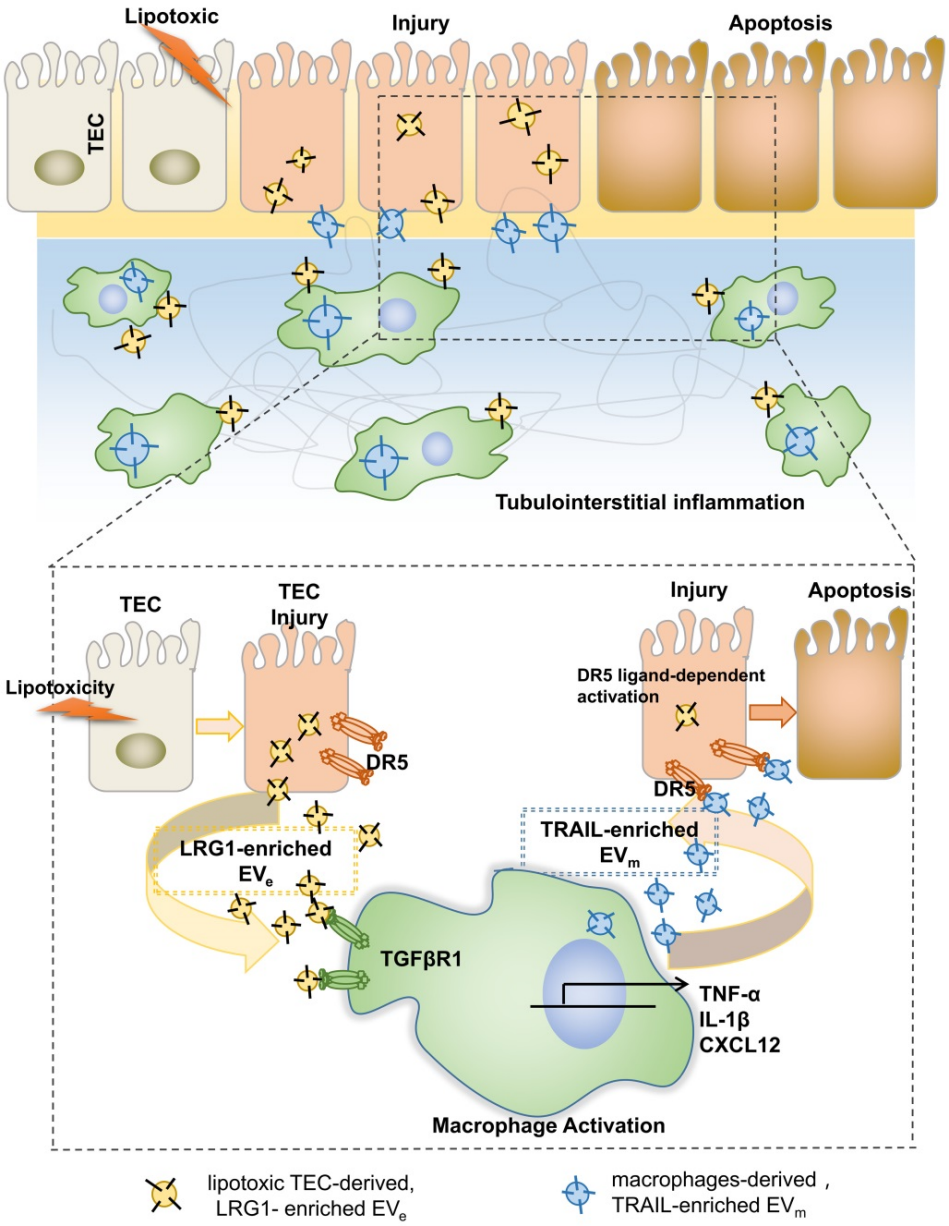
Tubular epithelial cell-to-macrophage forms a feedback loop via extracellular vesicle transfer promotes renal inflammation and apoptosis in diabetic nephropathy.

In summary, on the basis of our findings, we proposed a working model in Figure 8. During DN progression, LRG1-enriched, lipotoxic TEC-derived EV_e_ activated macrophages by a TGFβR1-dependent pathway and subsequently upregulated the expression of many inflammatory genes, thereby inducing DN inflammation and injury. Meanwhile, LRG1/TGFβR1 signaling also elevated TRAIL expression in macrophages, and TRAIL-enriched, macrophage-derived EV_m_ promoted apoptosis of TECs, which acted as a feedback loop in DN. Thus, our findings indicated a novel cell communication mechanism between tubular epithelial cells and macrophages in DN, which could be a potential therapeutic target.

## Supplementary Material

Supplementary figures and table.

## Abbreviations

T2DM: Type 2 diabetes mellitus
EV: extracellular vesicles
TECs: tubular epithelial cells
DN: diabetic nephropathy
LRG1: leucine-rich α-2-glycoprotein 1
DR5: death receptor 5
TNFRSF10B: factor receptor superfamily member 10b
TRAIL: tumor necrosis factor-related apoptosis-inducing ligand
LPC: lysophosphatidyl choline
HK-2: human kidney tubular epithelial cells
mTEC: mouse kidney tubular epithelial cells
NTA: nanoparticle tracking analysis
TSG101: tumor susceptibility gene 101
ARF6: adenosine diphosphateribosylation factor 6
BG: blood glucose
NLs: neutral lipids
ORO: Oil Red O
PAS: periodic acid-Schiff
GME: glomerular mesangial expansion
EPCAM: mouse primary epithelial cell adhesion molecule
TNF-α: tumor necrosis factor α
IL: interleukin
CXCL: chemokine (X-C motif) ligand
Cyto D: Cytochalasin D
