# Supplementary material for: Tubular epithelial cell-to-macrophage communication forms a negative feedback loop via extracellular vesicle transfer to promote renal inflammation and apoptosis in diabetic nephropathy
Source: Theranostics. 2022 Jan 1;12(1):324–39. doi: 10.7150/thno.63735 (PMC8690920; doi:10.7150/thno.63735)
Supplement: Supplementary file 1 — Supplementary figures and table. [file thnov12p0324s1.pdf]

Supplemental Table1.

Ingredients of HFD diet.

Ingredients	gm	kcal
Casein, 80Mesh	200	800
L-Cystine	3	12
Maltodextrin 10	125	500
Sucrose	68.8	275
Cellulose,BW200	50	0
Soybean Oil	25	225
Lard	245	2205
Mineral Mix S10026	10	0
DiCalcium Phosphate	13	0
Calcium Carbonate	5.5	0
Potassium Citrate,1 H_2O	16.5	0
Vitamin Mix V10001	10	40
Choline Bitartrate	2	0
FD&C Blue Dye#1	0.05	0
Total	773.85	4057

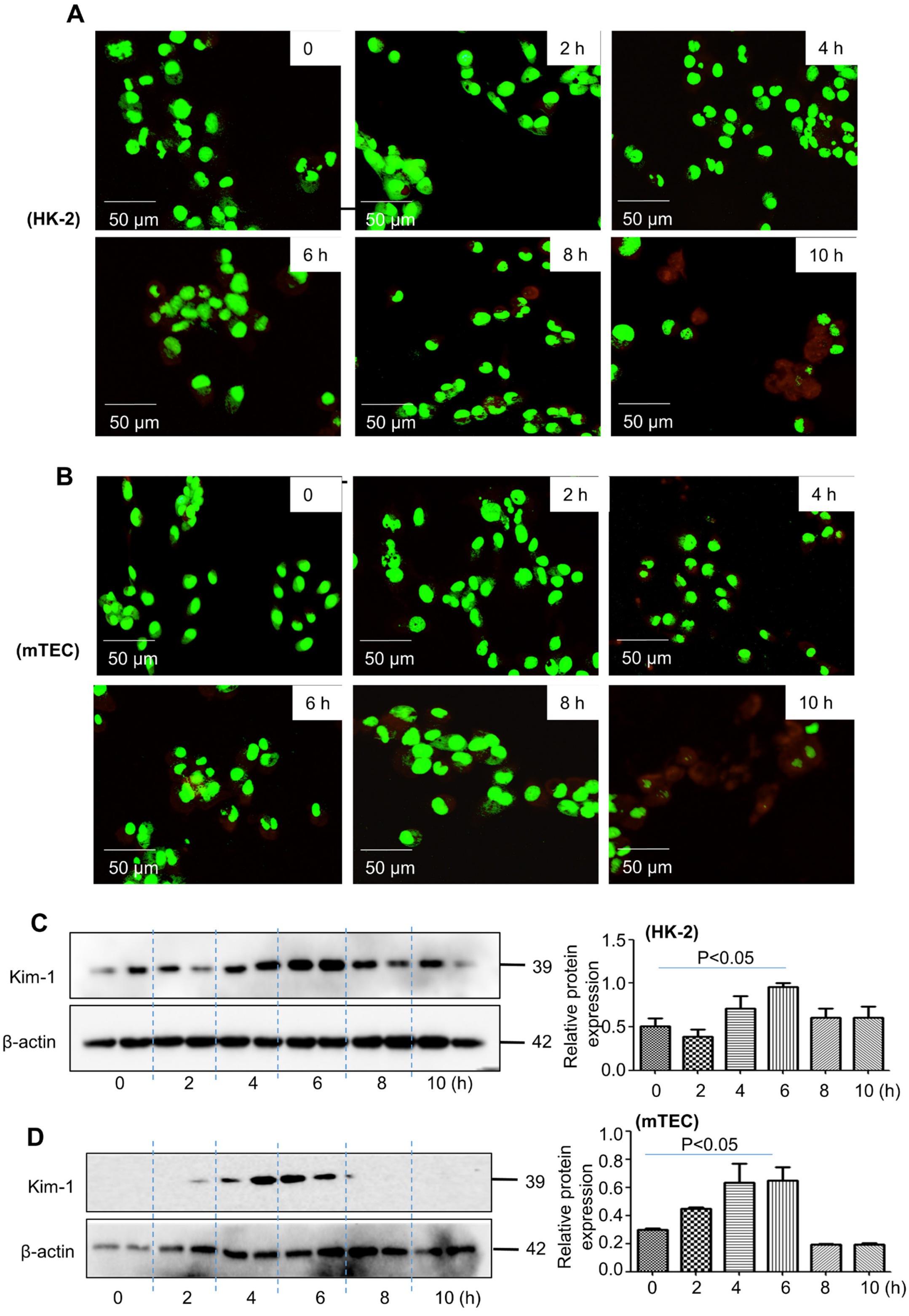
A
(HK-2)
0
2 h
4 h
6 h
8 h
10 h
50 μm
B
(mTEC)
C
Kim-1
β-actin
39
42
0 2 4 6 8 10 (h)
(HK-2)
P<0.05
Relative protein expression
1.5 1.0 0.5 0.0
D
(mTEC)
1.0 0.8 0.6 0.4 0.2 0.0

Supplementary Figure S1 Lipotoxicity induces release of tubular epithelial cells (TEC)-derived EV (EVe) in vivo and in vitro

(A) Expression of KIM-1 in HK-2 cells was detected by western blot.

(B) Expression of KIM-1 in mTEC was detected by western blot.

(C) Effects of various concentrations lysophosphatidyl choline (LPC) on apoptosis of HK-2 cells analysed by immunofluorescence staining.

(D) Effects of various concentrations lysophosphatidyl choline (LPC) on apoptosis of mTEC analysed by immunofluorescence staining.

Similar results were obtained in 3 independent experiments or in triplicate culture assays.

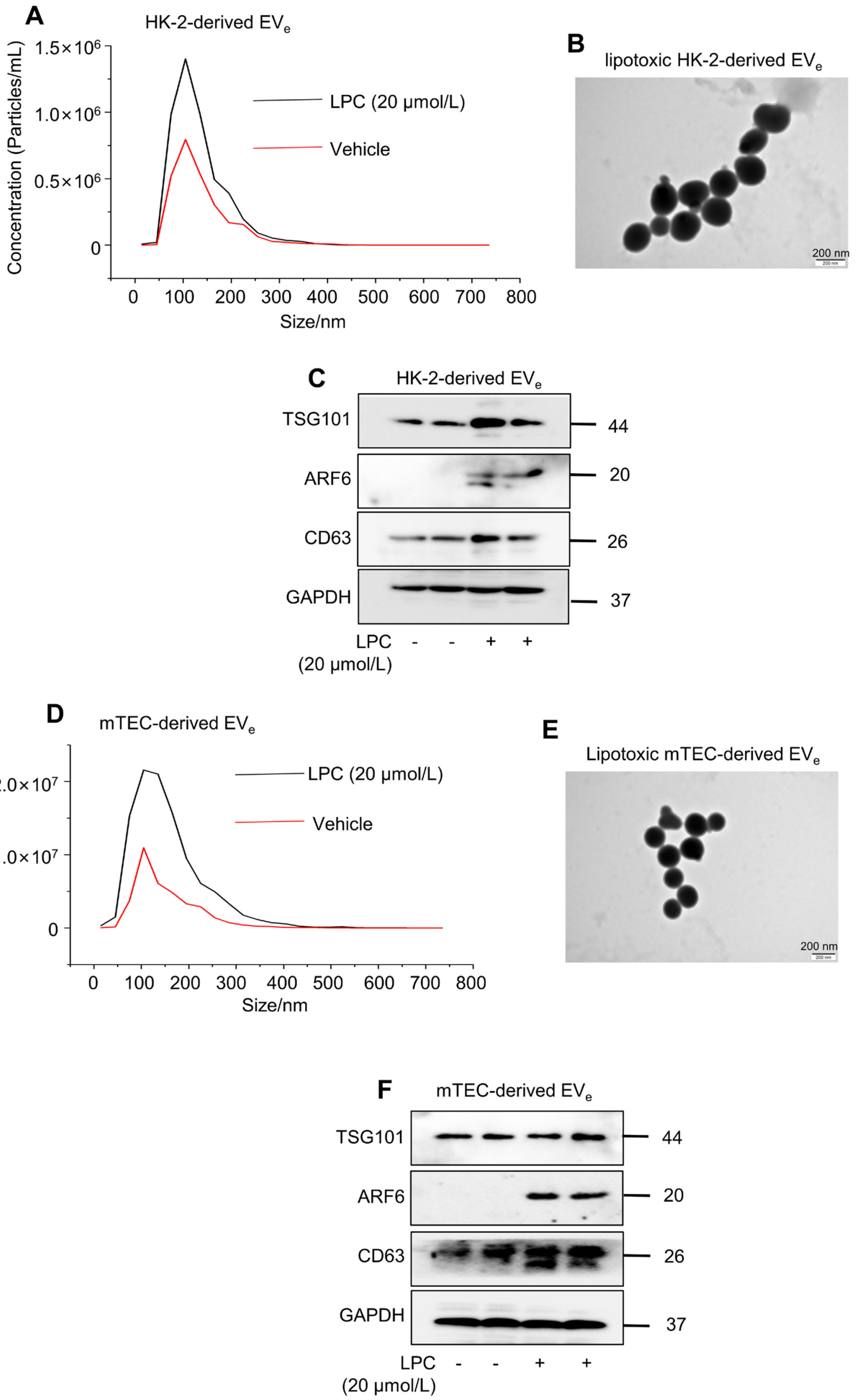
A
HK-2-derived EVe
Concentration (Particles/mL)
1.5×10^6
1.0×10^6
0.5×10^6
0
LPC (20 μmol/L)
Vehicle
0 100 200 300 400 500 600 700 800
Size/nm
B
lipotoxic HK-2-derived EVe
200 nm
C
HK-2-derived EVe
TSG101
ARF6
CD63
GAPDH
44
20
26
37
LPC - - + +
(20 μmol/L)
D
mTEC-derived EVe
2.0×10^7
1.0×10^7
0
LPC (20 μmol/L)
Vehicle
0 100 200 300 400 500 600 700 800
Size/nm
E
Lipotoxic mTEC-derived EVe
200 nm
F
mTEC-derived EVe
TSG101
ARF6
CD63
GAPDH
44
20
26
37
LPC - - + +
(20 μmol/L)

Supplementary Figure S2 Lipotoxicity induces release of tubular epithelial cells (TEC)-derived EV (EVe) in vivo and in vitro

(A) HK-2-derived EV_e representative image with nanoparticle tracking analysis (NTA).

(B) Transmission electron photomicrographs of lipotoxic HK-2-derived EV_e.

(C) Expression of TSG101, ARF6, CD63 and β-actin in HK-2-derived EV_e were detected by western blot.

(D) mTEC-derived EV_e representative image with nanoparticle tracking analysis (NTA).

(E) Transmission electron photomicrographs of lipotoxic mTEC-derived EV_e.

(F) Expression of TSG101, ARF6, CD63 and β-actin in mTEC-derived EV_e were detected by western blot.

Similar results were obtained in 3 independent experiments or in triplicate culture assays.
